# A Crimean Ghost Story

**Published:** 1893-01

**Authors:** 


### A CRIMEAN GHOST STORY.

During the Crimean war a captain in the army had a younger brother in the Seventh Royal Fusiliers, before Sebastopol, to whom he was much attached. One night he suddenly awoke in the bed and saw the figure of his brother kneeling in the room, looking anxiously and lovingly at him, through a light sort of phosphorescent mist. He noticed with horror a wound on the right temple of the head of the recumbent figure, from which a red stream flowed.

The face was of a waxy hue, but transparent looking, and so was the reddish mark. The narrator got up, went into the next room, called the members of his family and told them what he had seen. On the Monday following news was received of the storming of the Redan, and a fortnight later a friend brought the intelligence of the brother's death, he having been killed in the attack. The narrator adds that "both the colonel of the regiment and one or two officers, who saw the body, confirmed the fact that the appearance was much according to my description, and the death wound was exactly where I had seen it."

The precise time of the poor officer's death is uncertain, for the body was not found for thirty-six hours afterwards. The brother's presentiment of what had happened was, however, on the night of the day on which the Redan was stormed.

Another case, this time illustrating the transference of pain, is that of the wife of a well known landscape painter, who awoke in bed with a start one morning, feeling that she had had a severe blow on her mouth, with a distinct sense that her upper lip was bleeding. She applied her handkerchief to what seemed to be the injured part, but there was no blood, nor, on looking in the glass, was there any swelling. She took note of the hour (7 o'clock), went to bed again, and treated the matter as only a dream. Some little time afterwards her husband, who had been out in a sail-boat all the morning, returned, and the wife noticed that he had rather purposely sat further away from her than was usual. and every now and then put his handkerchief furtively up to his lip, in the way she had herself done earlier in the morning. Asking for an explanation she was told that "when I was sailing, a sudden squall came up, throwing the tiller suddenly around, and it struck me a bad blow in the mouth under the upper lip, and it has been bleeding a good deal and it won't stop." The hour, the husband said, must have been about 7. His wife adds that "I then told him what had happened to me, much to his surprise and to all who were with us at breakfast."

